# Rapid auditory processing of puretones is associated with basic components of language in individuals with autism spectrum disorders

**DOI:** 10.1016/j.bandl.2023.105229

**Published:** 2023-02-06

**Authors:** Carly Demopoulos, Brandon E. Kopald, Nitin Bangera, Kim Paulson, Jeffrey David Lewine

**Affiliations:** a University of California-San Francisco, Department of Psychiatry & Behavioral Sciences, 675 18th Street, San Francisco, CA 94107, United States; b University of California-San Francisco, Department of Radiology & Biomedical Imaging, 513 Parnassus Avenue, S362, San Francisco, CA 94143, United States; c University of California-San Francisco, Department of Neurology, 675 Nelson Rising, Lane, San Francisco, CA 94143, United States; d Mind Research Network, Pete & Nancy Domenici Hall, 1101 Yale Blvd. NE, Albuquerque, NM 87106, United States; e Center for Advanced Diagnostics, Evaluation and Therapeutics, CADET-NM, 1501 Indian School, NE, Albuquerque, NM 87102, United States; f University of New Mexico, Departments of Psychology and Neurology, 1 University Blvd. NE, Albuquerque, NM 87031, United States

**Keywords:** Autism Spectrum Disorder, Magnetoencephalography, Auditory processing, Communication, Speech, Language

## Abstract

The goal of this study was to identify the specific domains of language that may be affected by deficits in rapid auditory processing in individuals with ASD. Auditory evoked fields were collected from 63 children diagnosed with ASD in order to evaluate processing of puretone sounds presented in rapid succession. Measures of language and its components were assessed via standardized clinical tools to quantify expressive and receptive language, vocabulary, articulation, and phonological processing abilities. Rapid processing was significantly and bilaterally associated with phonological awareness, vocabulary, and articulation. Phonological processing was found to mediate the relationship between rapid processing and language. M100 response latency was not significantly associated with any language measures. Results suggest that rapid processing deficits may impact the basic components of language such as phonological processing, and the downstream effect of this impact may in turn impact overall language development.

## 1. Introduction

Deficits in communication are a defining feature of autism spectrum disorder (ASD). While the pathological processes that underlie these deficits are not fully understood, prior evidence has linked communication impairment to deficits in auditory processing. Indeed, abnormal auditory processing is a well-established finding among studies of individuals with ASD (Hitoglou et al., 2010; Z. J. Williams et al., 2020). In fact, several auditory processing anomalies have been identified as potential biomarkers of ASD and ASD symptomatology (Port et al., 2015). Studies have reported deficits in auditory filtering, (Alcántara et al., 2004; DePape et al., 2012; Tomchek et al., 2014) aberrant pitch perception (Bonnel et al., 2003; Heaton, 2003, 2005; Kargas et al., 2015; Mayer et al., 2014; O’Riordan & Passetti, 2006; Stewart et al., 2015), and abnormal auditory brainstem response (Demopoulos & Lewine, 2016; Klin, 1993; Russo et al., 2008; Russo, Nicol, et al., 2009). Deficits have been implicated in both the peripheral (Demopoulos & Lewine, 2016; Jure et al., 1991; Khalfa et al., 2001; Rosenhall et al., 1999) and cortical auditory processing systems, including absent signals (Edgar et al., 2014; Tecchio et al., 2003), anomalous oscillatory profiles (Edgar et al., 2013; Gandal et al., 2010; Wilson et al., 2007), atypical lateralization (Matsuzaki, Ku, et al., 2019; Matsuzaki, Kuschner, et al., 2019), reduced signal amplitude (Abdeltawwab & Baz, 2015; Ludlow et al., 2014; Russo, Zecker, et al., 2009; Z. J. Williams et al., 2020), impaired rapid processing (Demopoulos et al., 2015; Oram-Cardy et al., 2005), and delayed processing components (Abdeltawwab & Baz, 2015; Demopoulos et al., 2015; Edgar et al., 2013, 2014,2015; Gage et al., 2003; Gandal et al., 2010; Kasai et al., 2005; Matsuzaki et al., 2020; Oram Cardy et al., 2008, 2005; Roberts et al., 2010, 2011; Russo, Zecker, et al., 2009; Z. J. Williams et al., 2020). Recent work has indicated that these auditory response latency delays persist into adulthood for individuals with ASD (Matsuzaki et al., 2020).

Much of this prior work has used magnetoencephalography (MEG) to derive these indices of auditory processing because, in many ways, MEG is an ideal tool for studying auditory processing in this population. Specifically, MEG signal captures neuronal activity via continuous recording with excellent spatial and temporal resolution. Further, the MEG sensor array surrounds the head noninvasively and data is recorded silently, so there is minimal sensory discomfort to the participant and no confounding auditory stimulus produced by the scanner itself. Finally, MEG signal is sensitive to the tangentially oriented currents produced in the auditory cortex.

Several MEG studies have tied auditory processing abnormalities to communication impairment in ASD (Roberts et al., 2008). For example, language skills evaluated on the Clinical Evaluation of Language Fundamentals-4th Edition (CELF-4; Semel, Wiig, & Secord, 2003) were negatively associated with latency of mismatch fields (MMF; an index of pre-attentive change detection) for vowel sounds (Berman et al., 2016; Roberts et al., 2011) and puretones (Roberts et al., 2011) in individuals with ASD. M100 responses to puretones were found to be absent in children with ASD and concurrent language impairment assessed via the CELF-4 (Edgar et al., 2014). Another study found latencies of auditory M50 and M100 responses to puretones to be delayed in minimally verbal children with ASD, with “minimally verbal” defined via parent report of expressive vocabulary of fewer than 30 spontaneously and communicatively used words or phrases (Roberts et al., 2019). In a study that examined MEG auditory responses in relation to verbal intellectual abilities on the Wechsler Intelligence Scale for Children-Fourth Edition (WISC-IV), the M200 auditory response to puretones was negatively associated with WISC-IV Verbal Comprehension Index (VCI) scores (Demopoulos et al., 2017). Rapid auditory processing deficits, measured via a MEG rapid tone processing paradigm, were also identified in language-impaired children and adolescents with ASD (Oram-Cardy et al., 2005).

These studies have all related basic auditory processing functions to overall language abilities or verbal intelligence, assessed via either a multidimensional measurement tool such as the CELF-4 or the WISC-IV, or via parent-reported functional language usage. While these methods are appropriate for capturing many of the complexities of language impairment, they cannot provide detail regarding the precise nature of the relationship between basic auditory processes and verbal communication skills. It stands to reason that any difficulty in processing sounds accurately, discriminately, or in rapid succession would adversely affect one’s processing of speech (both one’s own or someone else’s) and result in impaired language development. Port et al. (2015) have suggested that basic components of language, such as phonological processing, may show a stronger relationship with auditory processing latency delays than broad assessment of complex language skills. This hypothesis is supported by neuroimaging studies demonstrating that speech is processed hierarchically, with initial processing of acoustic features in the dorsal superior temporal gyrus and phonological processing in the ventral superior temporal sulcus and middle temporal gyrus (L. Zhang et al., 2011). Despite this theoretical support, direct evidence is lacking for the hypothesis that basic auditory processing impairment is more strongly associated with basic components of language than overall language abilities. In the present study we evaluated the relationships between some of the most well-replicated electrophysiological findings in ASD (delayed auditory response latency and impaired rapid processing) and a range of clinical measures of verbal communication (i.e., expressive and receptive language, vocabulary, articulation, and phonological processing) to determine which specific language functions were associated with basic auditory processing abnormalities in a sample of children with ASD who presented with a wide range of communication abilities.

## 2. Methods

### 2.1. Participants

Participants were 63 English-speaking children (46 males, 17 females) ages 5–18 (M = 10.42, SD = 3.25) with a DSM-IV-TR diagnosis of ASD. Individuals diagnosed with Fragile-X, Tuberous Sclerosis or any comorbid neurological conditions other than epilepsy were excluded. Criteria for inclusion were: (1) DSM-IV-TR diagnosis of Autistic Disorder, Asperger’s Syndrome, or Pervasive Developmental Disorder—Not Otherwise Specified (PDD-NOS), as supported by data on the Autism Diagnostic Interview-Revised (ADI-R; Lord, Rutter, & Le Couteur, 1994) and Autism Diagnostic Observation Schedule (ADOS; Lord et al., 1989), (2) age within the specified range of 5–18 years, and (3) no contraindication for MEG or MRI such as braces or other permanent metal in the body. Participants who were taking medications were not asked to stop medications during study participation. Participants were taking anti-depressant (N = 11), stimulant (N = 14), antipsychotic (N = 9), anti-convulsant (N = 6), antihistamine (N = 7), sedative (N = 4), anxiolytic (N = 2), steroid inhaler (N = 2), bronchodilator (N = 1), beta blocker (N = 1), and cognition enhancing (N = 3) medications. Demographic data are presented in Table 1.

### 2.2. Procedures

Following an initial visit to obtain informed consent and assent, participants were scheduled for a diagnostic evaluation and two additional sessions for neuropsychological testing. Breaks and practice sessions were offered as needed, and when necessary, visits were broken up into shorter sessions to accommodate participant needs. Electrophysio-logical data were collected at a separate session following completion of the diagnostic and neuropsychological testing.

### 2.3. Measures

#### Diagnostic Assessment.

The diagnosis of ASD was confirmed according to DSM-IV-TR criteria through consensus diagnosis from the neuropsychology team under the supervision of a licensed clinical neuropsychologist. Diagnostic assignment was informed by information obtained from the ADOS, ADI-R, a neuropsychological history questionnaire, and relevant language and intelligence test performance. The ADI-R is an extensive diagnostic interview designed to elicit information that is relevant to the diagnosis of autism (Lord, Rutter, & Le Couteur, 1994). Psychometric studies of the ADI-R have indicated good discriminant validity (Rutter, LeCouteur, & Lord, 2003) and test–retest reliability ranging from 0.93 to 0.97 (Lord et al, 1993, 1994). The ADOS (Lord et al. 1989) is a semi-structured observational tool used to quantify behavior in relation to autism symptomatology. In a study of classification accuracy of the ADOS compared to consensus clinical diagnosis the ADOS effectively differentiated autism from non-spectrum disorders with specificities of 0.93–1.0 (Lord et al., 2000). Age-appropriate tests of intellectual abilities were administered to contextualize information relevant to diagnostic decisions. The Wechsler Intelligence Scale for Children-Fourth Edition (WISC-IV; Wechsler, 2003), the Wechsler Adult Intelligence Scale-Fourth Edition (WAIS-IV; Wechsler, 2008), or the Wechsler Preschool and Primary Scale of Intelligence-Third Edition (WPPSI-III; Wechsler, 2002) were administered to determine age-scaled full-scale intelligence quotients.

#### Assessment of Communication Abilities.

Language ability was assessed on the Clinical Evaluation of Language Fundamentals-Fourth Edition (CELF-4; Semel, Wiig, & Secord, 2003), a comprehensive language battery, to derive an overall age-scaled language quotient based on a normative sample along with indices of receptive and expressive language abilities. Expressive and receptive vocabulary were evaluated on the Expressive Vocabulary Test (EVT; Williams, 1997) and the Peabody Picture Vocabulary Test-3rd Edition (PPVT-3; Dunn, 1997), respectively. The EVT asks the examinee to identify the vocabulary word depicted in the picture. The PPVT-3 required the examinee to identify the picture from a choice of four options that best captures the stated vocabulary word. Articulation was assessed via the Sounds-In-Words subtest of the Goldman-Fristoe Tests of Articulation-2nd Edition (GFTA-2; Goldman & Fristoe, 2000), which requires the examinee to pronounce words with low vocabulary demands elicited by picture stimuli to evaluate accurate articulation of specific sounds. Finally, phonological processing abilities were evaluated on the Phonological Awareness composite score of the Comprehensive Test of Phonological Processing (CTOPP; Rashotte, Torgesen, & Wagner, 1999), which assesses awareness and access to the phonological structure of spoken language.

#### Assessment of Cortical Auditory Processing.

Auditory evoked fields were collected during a MEG Rapid Auditory Processing Task. This task is designed to evaluate the ability of the brain to process sounds presented in rapid succession. Three separate conditions of this task were presented to assess (1) response to single puretone sounds, (2) rapid processing via response to two different pure-tone sounds (1000 Hz and 2000 Hz) and (3) sensory gating (response to pairs of the same tones). The focus of this study is on condition 2: response to two different tone pairs. Data were averaged from 150 trials consisting of two different 50 ms-long tones (500 Hz followed by 1000 Hz or 1000 Hz followed by 500 Hz) presented 300 ms apart with an inter-trial-interval of 2000 ms. A 10% hamming window was applied to each individual tone. To ensure that stimuli were audible to all participants, hearing thresholds were captured via puretone audiometry and stimuli were presented at peak amplitude of 75 dB SPL through loudspeakers, which was at least 30 dB above hearing thresholds. Prior research has demonstrated that a reliable AEP can be evoked at a stimulus intensity within 20 dB of the PTA hearing threshold (Misale et al., 2020).

Data were collected using a 306-channel biomagnetometer system (VectorView, Elekta, Oy, Helsinki) with participants oriented in a supine position to stabilize head position. The system consists of an array of planar gradiometers and magnetometers, distributed at 102 spatial positions with one magnetometer and a pair of orthogonal planar gradiometers at each location. Prior to testing, four small coils were placed on the head. A 3D-digitizer was used to define a head-centered coordinate frame (using the nasion and *peri*-auricular points), and the position of the coils within the frame. During testing, the coils were energized and localized by the sensor array in a manner that defines the position of each sensor relative to the head.

Because the MEG task involved only passive exposure to auditory stimuli, participants were allowed to watch a movie without sound while in the scanner as reported in prior studies of auditory processing in this population (Edgar et al., 2013, 2014; Oram Cardy et al., 2008; Roberts et al., 2008, 2012). The study team also included advanced clinical psychology graduate students and postdoctoral fellows who have extensive training and experience in working with children with ASD who present with a range of functional abilities, including nonverbal children and children who present with challenging behavior. The combination of the minimal task demands, provision of silent video entertainment, and the clinical skills of the study team allowed for inclusion of the representative range of participants in this dataset.

Raw data were collected with a 1000 Hz digitization rate with a 0.1 – 300 Hz bandwidth. Artifacts from more proximal noise sources such as eye blink and heartbeat were removed using signal space projections (SSP) defined by visual inspection of the data, and signal space separation with temporal extension (Taulu & Hari, 2009) was used to remove artifacts from distal noise sources. Single trial epochs with a baseline of 250 ms and a post-stimulus duration of 1000 ms were then generated and averaged. Prior to averaging, individual epochs were rejected if they contained large artifacts (>2pT) or evidence of residual eye blinks, eye movements, or head movements upon visual inspection. All data sets retained a minimum of 130 out of the 150 trials. Average responses were baseline corrected and subjected to additional band-pass filtering (1–30 Hz). Exploratory analyses using dipole modeling of the M100 response in each hemisphere indicated that differences in source localization for the 500 and 1000 Hz tones were small (less than 5 mm) and inconsistent across participants. Thus, 500/1000 Hz and 1000/500 Hz trials were averaged to maximize the signal to noise ratio.

For each hemisphere, for each subject, the M100 was identified as the first post-stimulus magnetic peak associated with field pattern consistent with a negative evoked potential at Cz. All but 6 subjects had simultaneous multi-channel EEG, which allowed for confirmation that the identified M100 was indeed the neuromagnetic counterpart of the EEG identified N1 response. In each hemisphere, a dipole source was placed in the temporal lobe. Using the Neuromag Xfit program, its position and orientation were optimized on a case-by-case basis over a 50 ms window spanning the peak latency of the corresponding hemisphere’s M100 response. A spherical head model was used in the calculations, with simultaneous optimization of left and right hemisphere dipole parameters. The resultant dipole model was then held fixed and, for each participant, source waveforms for the auditory response to the different tone pairs were generated by ‘passing’ that condition’s average evoked response through the individual participant’s fixed model. This is the equivalent of the source space projection method described by Tesche et al. (1995) and Wilson et al. (2008).

**Fig. 1.**
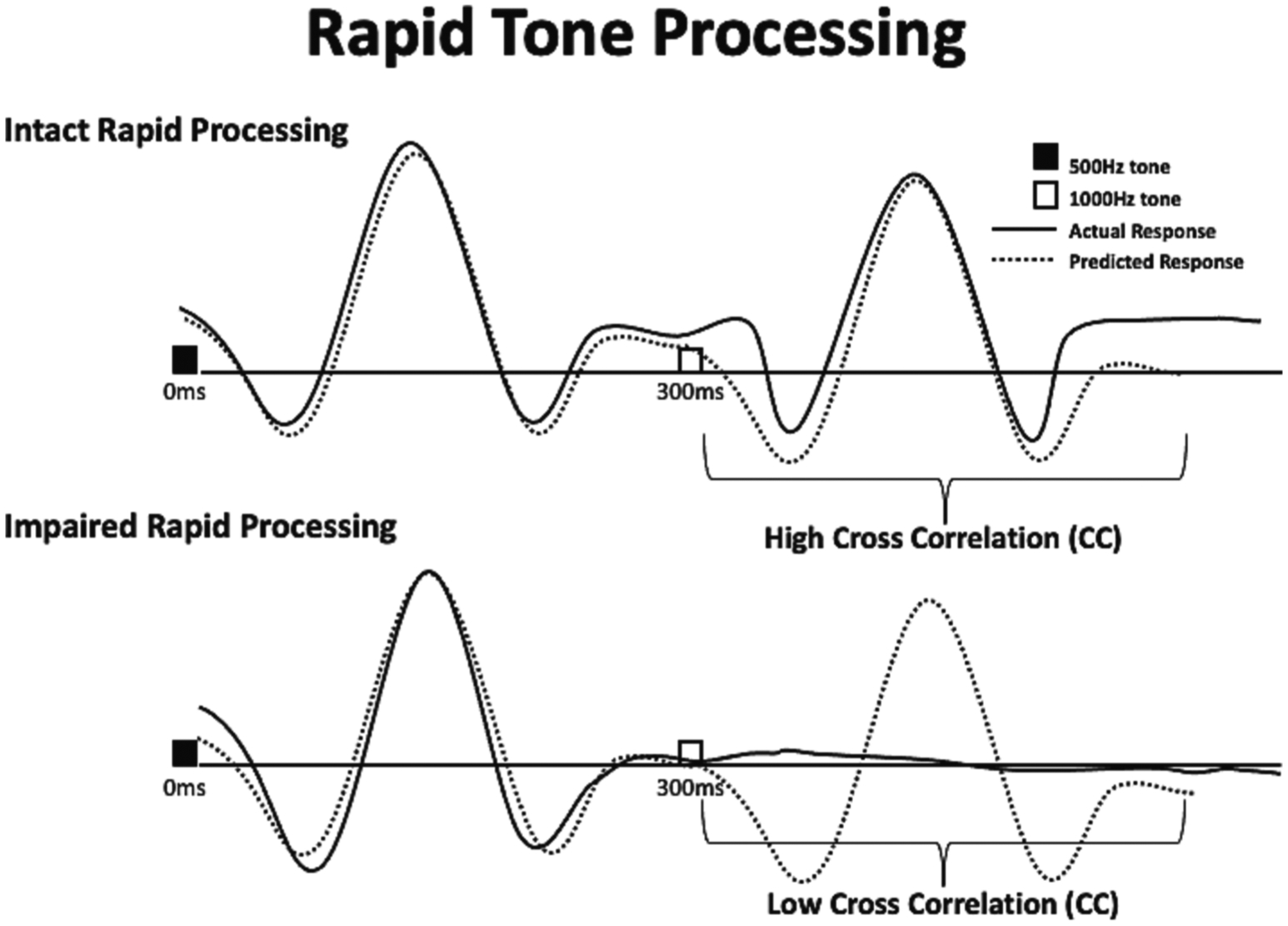
Schematic of Rapid Auditory Processing Index. Participant responses to single tones at 0–300 ms were projected onto the 300–600 ms time window to generate a predicted waveform (dotted lines). Thus, the dotted line represents a predicted response to both tones based on the assumption of identical first and second tone responses. This predicted waveform was generated as a standard of comparison for intact rapid processing for each participant. Specifically, the individual participant’s predicted waveform was compared via cross correlation against their actual response (solid lines) to the two tones presented in rapid succession (the first at 0 ms and the second at 300 ms). High agreement at 300–600 ms between predicted and actual waveforms in the top drawing indicates intact rapid processing, as there is clear and consistent response to both tones. Low agreement between waveforms in the bottom drawing indicates impaired rapid processing, as the response to the second tone is absent.

To index rapid processing we focused on the overall quality of the response to the second tone (Oram-Cardy et al., 2005) in the discordant tone pairs. First, a predicted waveform was created for each participant for comparison to their actual waveform. The predicted waveform was created by adding two waveforms. Waveform 1, which is the response to a single tone, was shifted forward in time by 300 ms with zero-fill for the shift and truncation at the end to create waveform 2. The two waveforms are then added to create an idealized waveform for a paired tone presentation where the responses to the first and second tone are physiologically identical with the second response superimposed on the first. No smoothing was performed on the final predicted waveform. Zero lag cross correlation coefficients (CCs) were then calculated to measure agreement between the participant’s auditory responses waveform and the predicted waveform in the 300–600 ms window. CC analyses were conducted using SPSS Version 20. The amplitudes of the right and left source waveforms were extracted in 5 ms steps. Response waveforms were then compared within each hemisphere’s 300–600 ms time window to yield separate CC values for right and left hemisphere responses. Higher CC values indicate greater agreement and lower values indicating poorer agreement between waveforms. For individuals with intact rapid processing, when two different tones are presented in rapid succession (i.e., 300 ms apart) the resultant waveform demonstrates two strong responses (one to the first, and one to the second tone). Thus, high agreement (reflected in a high CC) between the actual and the predicted waveform in the 300–600 ms window would be indicative of intact rapid auditory processing. In contrast, if rapid processing were impaired, the waveform would be characterized by poor quality of response to the second tone, reflected by low agreement between actual and predicted waveforms and a corresponding low CC value (Fig. 1).

#### Data Analytic Plan.

Based on previous research on the relationship between basic auditory processing and language impairment in individuals with ASD (Port et al., 2015), we hypothesized that MEG indices of rapid auditory processing would be significantly associated with basic language functions (i.e., vocabulary, articulation, phonological processing), but that weaker associations would be found between cortical auditory processing and overall receptive and expressive language abilities measured via the CELF-4. To test these hypotheses, Pearson correlations were performed between MEG cortical measures of left- and right-hemisphere rapid auditory processing and M100 response latencies and norm-referenced standard scores derived for all communication measures, including the CELF-4 (receptive and expressive language), the GFTA-2 (articulation), PPVT-3 (receptive vocabulary), EVT (expressive vocabulary), and CTOPP (phonological processing). Because CC values were used to quantify cortical rapid auditory processing, these scores were transformed to Fisher’s Z before being subject to further analysis in order to correct for the non-normality of the r distribution.

## 3. Results

**Fig. 2.**
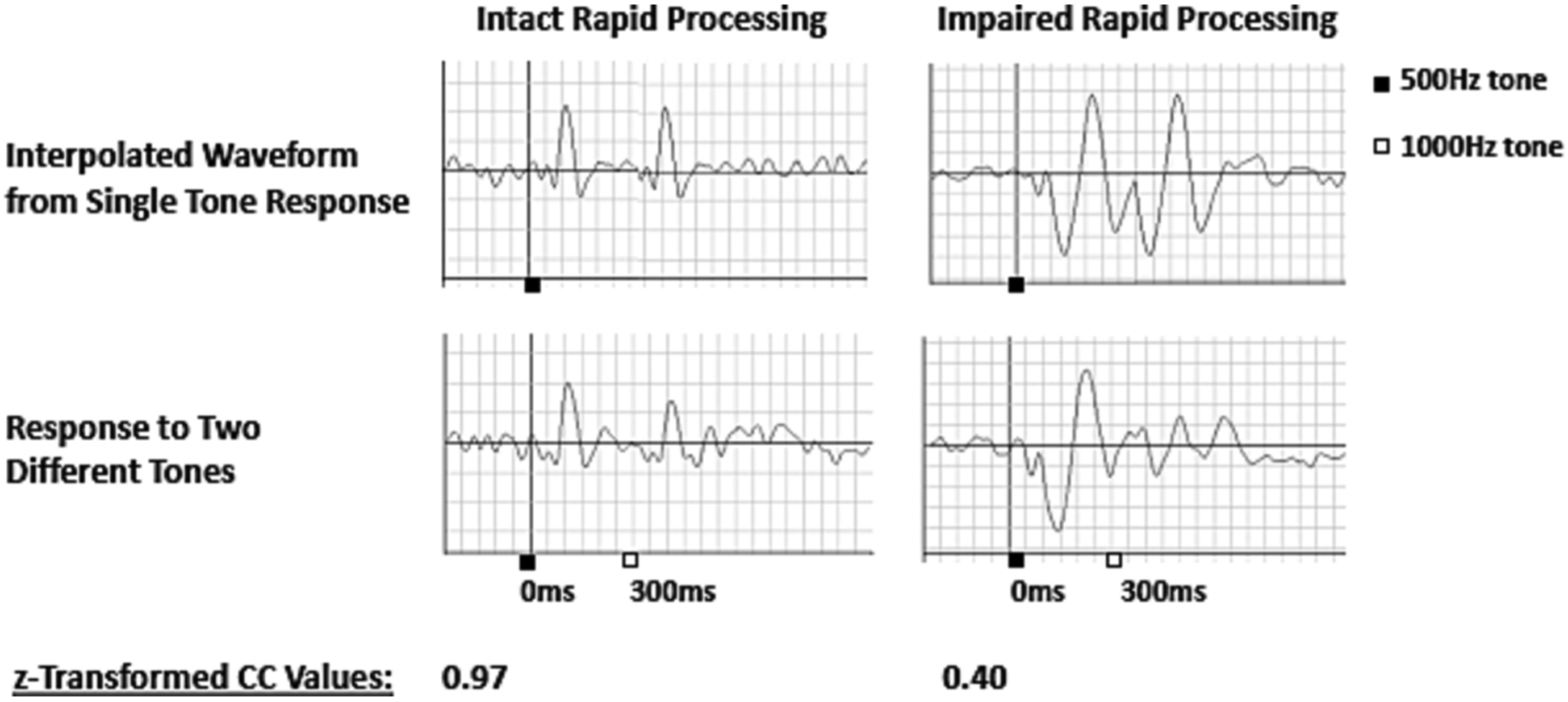
Example Waveforms and Associated CC Values for Intact (left) and Impaired (right) Rapid Processing. The waveforms on the left demonstrate the high agreement between predicted (top) and actual response (bottom) waveforms in a participant with intact rapid processing with a corresponding high z-transformed CC value. A response is clearly identifiable approximately 100 ms after the presentation of each tone. In contrast, the waveforms in the right column demonstrate poor agreement between the predicted (top) and actual response (bottom) waveforms, with a corresponding low z-transformed CC value, indicating impaired rapid processing. This participant’s initial response is delayed and prolonged, such that the participant is still processing the first tone at the time the second tone is presented.

M100 latencies were delayed bilaterally, with M = 161.949, SD = 54.804 and a range of 90–260 ms in the left hemisphere and M = 159.983, SD = 56.970 and a range of 95–300 ms in the right hemisphere. Fig. 2 illustrates how cross correlation values were derived for two participants with intact and impaired rapid processing, respectively. Actual responses were compared against dual response waveforms predicted from projection of initial tone response onto the second tone response window. Z-transformed cross correlation values for the 300–600 ms time window ranged from values with low agreement (z = −1.12 for left hemisphere and z = −0.51 for right hemisphere) to high agreement (z = 1.50 for left hemisphere and z = 1.60 for right hemisphere), indicating that our sample incorporated a broad spectrum of function with regard to rapid processing. Paired samples t-tests indicated no significant within-participant differences between rapid processing in the right (M = 0.632, SD = 0.398) versus left hemisphere (M = 0.601, SD = 0.467), t(62) = −0.678, p =.500) nor between right (M = 159.983, SD = 56.970) and left (M = 161.95, SD = 54.804) M100 latency t(58) = 0.664, p =.509. There were no gender differences in rapid processing for either hemisphere (RH t(61) = 0.874, p =.386, LH t (61) = 0.807, p =.432) and rapid processing was not significantly associated with age (RH r = 0.233, p =.067; LH r = 0.213, p =.095).

**Fig. 3.**
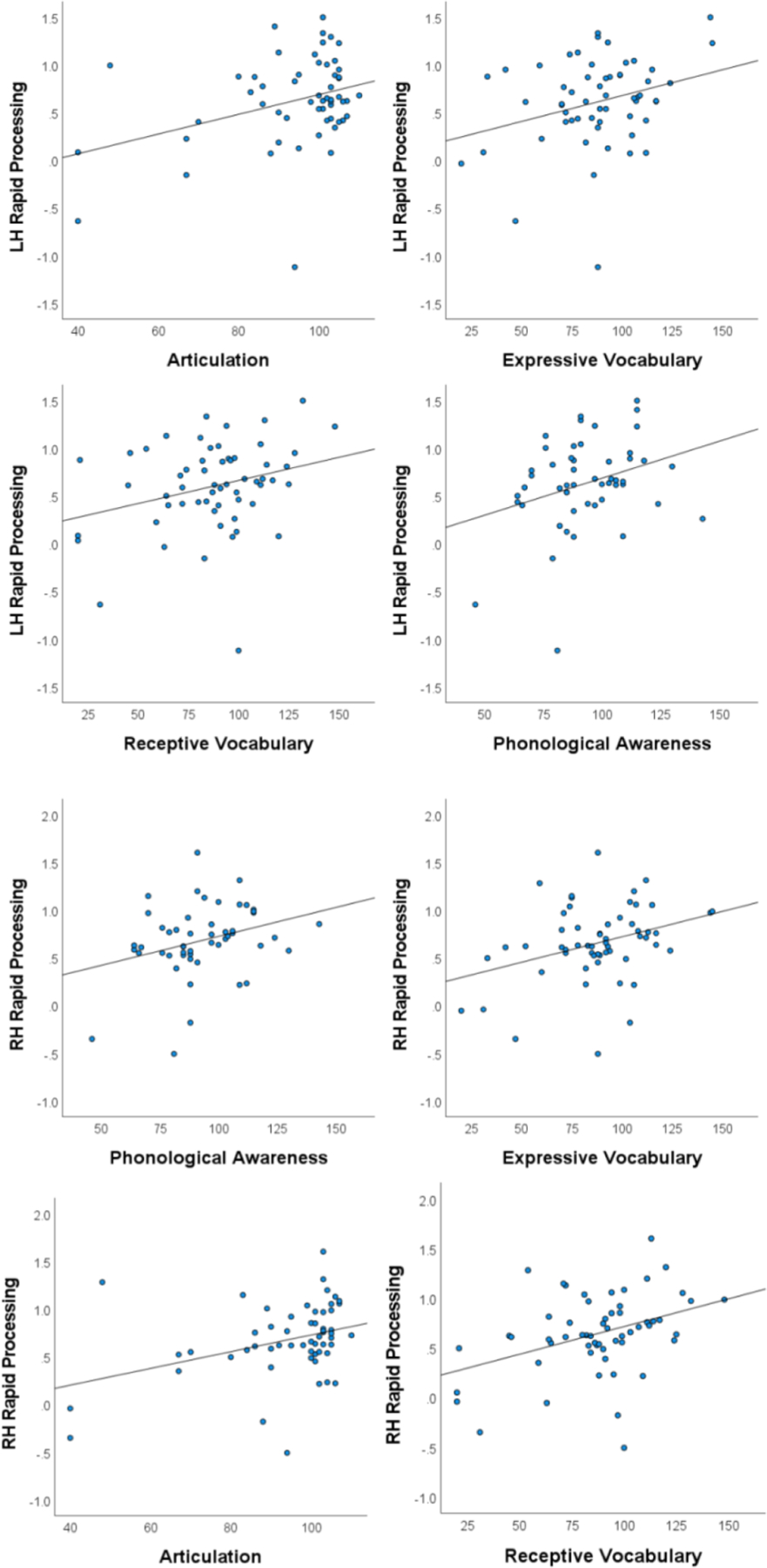
Scatterplots of Associations Between Rapid Processing and Language Measures. Scatterplots illustrate the multiple associations between the rapid processing indices and basic components of language bilaterally.

Because two analyses (left hemisphere and right hemisphere) were performed for each hypothesized relationship to language skills, a Hochberg FDR correction was computed to adjust for type 1 error among the two analyses. Following this correction, rapid auditory processing was significantly and bilaterally associated with phonological awareness (N = 51; LH: r = 0.306, p =.029; RH: r = 0.299, p =.033), receptive (N = 58; LH: r = 0.285, p =.030; RH: r = 0.384, p =.003) and expressive vocabulary (N = 57; LH r = 0.293, p =.027; RH: r = 0.339, p =.010), and speech articulation (N = 56; LH: r = 0.349, p =.008; RH: r = 0.358, p =.007). These associations are represented in the scatterplots presented in Fig. 3. Neither receptive (N = 56; LH: r = 0.149, p =.272; RH: r = 0.159, p =.241) nor expressive language (N = 55; LH: r = 0.190, p =.165; RH: r = 0.156, p =.254) were associated with rapid processing in either hemisphere.

Significant relationships between measure of communication and M100 response latency were not identified in either hemisphere. Specifically, M100 latency was not associated with expressive (CELF-IV; N = 53; LH: r = 0.218, p =.118; RH: r = 0.128, p =.326) or receptive language (CELF-IV; N = 54; LH: r = 0.165, p =.234; RH: r = 0.135, p =.330), expressive (EVT; N = 54; LH: r = 0.149, p =.283, RH: r = 0.074, p =.597) or receptive vocabulary (PPVT-3; N = 55; LH: r = 0.165, p =.229; RH: r = 0.117, p =.349), phonological processing (CTOPP; N = 50; LH: r = 0.080, p =.579; RH: r = 0.033, p =.821), or articulation (GFTA; N = 54; LH: r = 0.076, p =.587; RH: r = 0.014, p =.918).

Given that an association was identified between cortical rapid auditory processing and phonological processing, but not between cortical rapid processing and expressive or receptive language abilities, post hoc mediation analyses were performed in SPSS 27 to evaluate whether phonological processing ability mediates the relationship between rapid processing and language abilities. For this analysis, linear regression analyses were performed to (1) estimate the direct effect of rapid processing on phonological processing (Model A), and (2) estimate the direct effect of rapid processing and phonological processing on language performance (Model B). Next, the unstandardized beta weight/standard error for rapid processing in regression Model A was compared to the unstandardized beta weight/standard error for phonological processing in regression Model B via a Sobel Test (Sobel, 1982). Finally, the indirect effect was computed by multiplying the beta coefficient for the path from rapid processing to phonological processing in Model A by the beta coefficient for the path from phonological processing to language in Model B. Separate mediation analyses were performed following these steps for right and left hemisphere rapid processing with expressive and receptive language scores each as dependent variables. Results indicated that phonological processing was found to mediate the effect of rapid auditory processing on receptive (LH: z = 2.103, p =.036, with a point estimate of the mediated effect, αβ = 10.643; RH: z = 2.062, p =.039, αβ = 13.042) and expressive language performance (LH: z = 2.077, p =.038, αβ = 11.102; RH: z = 2.045, p =.041, αβ = 13.96).

## 4. Discussion

The goal of the current study was to investigate the specific domains of verbal communication that are impacted by deficits in rapid processing of basic auditory information for children with ASD. These domains were assessed using clinical measures of expressive and receptive language, vocabulary, phonological processing, and articulation and were examined in relation to MEG indices of rapid auditory processing and response latency for the first time in individuals with ASD.

Generally consistent with previous MEG studies identifying abnormal auditory processing in individuals with ASD (Demopoulos et al., 2015; Edgar et al., 2013; Jenkins et al., 2016; Oram-Cardy et al., 2005; Oram Cardy et al., 2008; Roberts et al., 2008, 2011,2012; Schmidt et al., 2009), we found significant correlations between (a) quality of rapid processing of basic auditory information (i.e., pure-tone sounds) bilaterally and (b) phonological processing, receptive and expressive vocabulary, and speech articulation. Previous work examining MEG measures of rapid processing in individuals with ASD reported that rapid processing was impaired in those with language impairment (Oram-Cardy et al., 2005). In the present study, however, rapid tone processing did not show a significant direct relationship to CELF-4 Expressive and Receptive Language Index scores. There are several possible reasons for the failure to replicate this direct relationship. First, the current study employed a novel methodological paradigm, using different paired pure-tones rather than identical paired pure-tones, which may account for the discrepancies with previous research findings. Specifically, Oram-Cardy et al. (2005) measured response to two 1000 Hz tones presented in rapid succession. Brain response to identical paired tones presented in rapid succession allows for measurement of the sensory gating phenomenon, in which the redundant sensory information (second presentation of the identical tone) produces a reduced cortical response amplitude relative to the initial tone. Sensory gating was not specifically assessed in Oram-Cardy et al. (2005), as relative amplitudes of first and second tones were not reported. Instead, presence or absence of specific auditory response components was examined in this study. Nevertheless, the sensory gating phenomenon indicates that the brain responds differently to novel (as in the current study) as opposed to redundant sensory information (as in the Oram-Cardy study). In the present study sensory gating could not be assessed, as it was found that several participants had impaired rapid processing of the second tone, such that the gating response could not be reliably measured without being potentially confounded with rapid processing impairment.

There are, however, several other methodological differences that indicate that the findings in these two studies are not directly comparable. Most notably, group differences were examined in children who scored in the average or higher range versus below average range on the CELF-IV and/or CTOPP. Thus, it is unclear if these group differences would have been identified in groups stratified by CELF-IV scores alone, or if phonological processing measured via the CTOPP was driving the difference in rapid processing identified by Oram-Cardy et al. In fact, phonological representations have been hypothesized to impact processing of basic acoustic information via a top down process (L. Zhang et al., 2011). The results of the present study identified a significant mediating effect of phonological processing on the relationship between rapid processing and language. These results suggest that deficits in rapid processing of basic auditory information may impact phonological processing, and the downstream effect of this impact may, in turn, impact language development. This interpretation is consistent with prior research describing a hierarchical organization to speech processing (L. Zhang et al., 2011), which would suggest that impairment in rapid processing at the initial acoustic level in the superior temporal gyrus subsequently impacts phonological processing in the superior temporal sulcus and middle temporal gyrus. Studies examining dynamic functional connectivity in these regions during speech processing are needed to examine these network dynamics.

The present study suggests that the relation of rapid processing to overall language skills is weaker than its relation to the basic components of language. This weaker association may be impacted by the relatively stronger contribution of other social and cognitive factors that impact overall language skill development, diminishing the effect of basic auditory processing. This interpretation is broadly consistent with the conclusions of Port et al. (2015), who suggested that, while basic auditory response delays may underly language impairment indirectly, a direct association between auditory response latency and overall language performance has not been established. In the present study, however, M100 latency was neither associated with overall language skills nor basic language components. While this null result should be interpreted with caution, as the present study was only powered to detect medium to large effects, the stronger associations identified between the basic language components and rapid processing of puretone sounds may suggest that previous studies associating latency delays with overall language abilities reflect an indirect link between the two. Indeed, previous research has demonstrated abnormalities in basic auditory processing, such as mismatch response latency, may be present as early as 12 months in children at risk for ASD (Riva et al., 2018). Further, these response delays were associated with expressive vocabulary at 20 months of age, even before more advanced language skills have developed.

Aside from these methodological differences, there are several other potential explanations for the lack of a direct relationship between rapid processing and overall receptive and expressive language performance. For example, prior research has demonstrated greater impairment in orienting to speech versus non-speech sounds in participants with ASD (Lepistö et al., 2005). Thus, is it possible that our rapid tone processing paradigm may not have targeted the specific process associated with expressive and receptive language skills and that a paradigm employing speech sounds would be necessary to detect this effect. Other studies, however, have reported impairment in processing both speech and non-speech sounds in individuals with ASD (J. Zhang et al., 2019). Future studies characterizing the processing of speech sounds and their relations to a range of measures of language and its components are necessary to clarify these distinctions.

### 4.1. Limitations and Future directions

There are several limitations of the present study that must be acknowledged. First, musical background of the participants was not assessed, and musical training can have an impact on pitch processing. Another limitation is that the age range included in this sample results in a lack of continuity across ages for the subtests used to derive the expressive and receptive language index scores. Thus, these constructs, while generally more reliable than individual subtest score, were not consistently derived across ages.

Another limitation of the present study was that sensory gating (suppression of evoked cortical response to a redundant stimulus) was not able to be examined in relation to language abilities despite availability of data from a same tone pair condition. Specifically, because auditory response delays and impaired rapid processing (e.g., poor quality or absent response to the second tone) were identified in this sample, sensory gating, which is quantified through characterization of the second response to same tone pairs, could not be reliably measured in this sample. Future studies with a large sample of participants with ASD who have intact rapid processing are needed to reliably evaluate the relationship between sensory gating and performance on a range of measures of language and its elemental components in this population. Further, additional research is necessary to understand the associations between other forms of auditory processing differences and language functioning in individuals with ASD. Specifically, studies with well-characterized large sample sizes who include individuals with a broad range of language abilities are needed. These studies should examine processing of a broad spectrum of auditory functions in order to isolate the specific auditory processes underlying the clinical manifestation of language and communication impairments in ASD and related disorders.

## 5. Conclusions

The present study provides further evidence of the association between rapid auditory processing and language functioning demonstrated in a prior study that classified language functioning according to performance on overall language or phonological processing abilities. The results of this study suggest that this relationship is driven by phonological processing abilities, as phonological processing was found to mediate the relationship between rapid processing and overall language performance, whereas a direct relationship between rapid processing and overall language index scores was not identified. Thus, the present study provides new insight into a mechanistic account of how rapid processing impairment may impact language via disruption of phonological processing.

## Figures and Tables

**Table 1:** Participant demographics.

	M	SD	Range
FSIQ	83.824	22.280	46–136
Language			
Expressive	79.546	27.125	45–132
Receptive	80.732	24.362	45–131
Vocabulary			
Expressive	87.807	25.004	20–145
Receptive	87.328	27.394	20–148
Phonological Proc.	93.098	18.637	46–143
Articulation	94.196	15.804	40–110
Gender (N)			
	Male	46	
	Female	17	
Ethnicity (N)			
	Caucasian	43	
	Hispanic	7	
	Asian	3	
	African American	3	
	Multiracial	6	
	Other	1	
